# Reliabilities of estimated breeding values in models with metafounders

**DOI:** 10.1186/s12711-023-00778-2

**Published:** 2023-01-23

**Authors:** Matias Bermann, Ignacio Aguilar, Daniela Lourenco, Ignacy Misztal, Andres Legarra

**Affiliations:** 1 Department of Animal and Dairy Science, University of Georgia, Athens, GA USA; 2 Instituto Nacional de Investigación Agropecuaria (INIA), Montevideo, Uruguay; 3 GenPhySE, INRAE, ENVT, Université de Toulouse, 31326 Castanet-Tolosan, France; 4 Present Address: Council on Dairy Cattle Breeding, Bowie, MD 20716 USA

## Abstract

**Background:**

Reliabilities of best linear unbiased predictions (BLUP) of breeding values are defined as the squared correlation between true and estimated breeding values and are helpful in assessing risk and genetic gain. Reliabilities can be computed from the prediction error variances for models with a single base population but are undefined for models that include several base populations and when unknown parent groups are modeled as fixed effects. In such a case, the use of metafounders in principle enables reliabilities to be derived.

**Methods:**

We propose to compute the reliability of the contrast of an individual’s estimated breeding value with that of a metafounder based on the prediction error variances of the individual and the metafounder, their prediction error covariance, and their genetic relationship. Computation of the required terms demands only little extra work once the sparse inverse of the mixed model equations is obtained, or they can be approximated. This also allows the reliabilities of the metafounders to be obtained. We studied the reliabilities for both BLUP and single-step genomic BLUP (ssGBLUP), using several definitions of reliability in a large dataset with 1,961,687 dairy sheep and rams, most of which had phenotypes and among which 27,000 rams were genotyped with a 50K single nucleotide polymorphism (SNP) chip. There were 23 metafounders with progeny sizes between 100,000 and 2000 individuals.

**Results:**

In models with metafounders, directly using the prediction error variance instead of the contrast with a metafounder leads to artificially low reliabilities because they refer to a population with maximum heterozygosity. When only one metafounder is fitted in the model, the reliability of the contrast is shown to be equivalent to the reliability of the individual in a model without metafounders. When there are several metafounders in the model, using a contrast with the oldest metafounder yields reliabilities that are on a meaningful scale and very close to reliabilities obtained from models without metafounders. The reliabilities using contrasts with ssGBLUP also resulted in meaningful values.

**Conclusions:**

This work provides a general method to obtain reliabilities for both BLUP and ssGBLUP when several base populations are included through metafounders.

## Background

In the traditional animal model, animals with unknown parents are assumed to be unrelated and to belong to the same base population. This base population is assumed to be of infinite size and with an average breeding value equal to zero. Under this assumption, using the correct model specification and with a complete pedigree, estimation of the breeding values $\left(\mathbf{u}\right)$ and variance components is unbiased [1, 2]. However, pedigrees are incomplete in most livestock populations, in which case genetic groups or unknown parent groups (UPG) are typically used to handle incomplete pedigrees and unrecorded selection [3]. From the early stages of their implementation, UPG were fitted as fixed effects [3–5], resulting in the final breeding value ${\mathbf{u}}^{*}=\mathbf{Q}\mathbf{g}+\mathbf{u}$ to be a weighted sum of the fixed group effects $\left(\mathbf{Q}\mathbf{g}\right)$ and a random deviation $\left(\mathbf{u}\right)$ [3]. As a result, the final breeding values (and their estimates) do not have an explicit base population, therefore, estimated breeding values (EBV) are not estimable, although their contrasts are [3]. Moreover, using fixed UPG is not justified from a quantitative genetics perspective because it assumes that the genetic variance is not changed by drift or selection [6], in other words, it assumes that UPG are of infinite size and not related to each other.

The reliability of an individual EBV can be defined as the squared correlation between the true and estimated breeding value over repeated conceptual sampling: $re{l}_{i}={r}^{2}\left(\widehat{u},u\right)$. Reliabilities can be obtained as $re{l}_{i}=1-\frac{\text{Var}\left({\widehat{u}}_{i}-{u}_{i}\right)}{\text{Var}\left({u}_{i}\right)}$ [7], where $\text{Var}\left({\widehat{u}}_{i}-{u}_{i}\right)$ is the prediction error variance of ${\widehat{u}}_{i}$, which can be obtained from the inverse of the coefficient matrix of the mixed model equations (MME) [8]. However, the expression $re{l}_{i}=1-\frac{\text{Var}\left({\widehat{u}}_{i}-{u}_{i}\right)}{\text{Var}\left({u}_{i}\right)}$ does not hold when UPG are fitted as fixed effects because then the covariance between true and estimated effects is not equal to the variance of the estimated effects [7]. Instead, when UPG are fitted as fixed effects, the reliability can be defined as ${r}^{2}\left({\widehat{u}}^{*},{u}^{*}\right)={r}^{2}(\mathbf{q}\widehat{\mathbf{g}}+\widehat{u},\mathbf{q}\mathbf{g}+u)$, where $\mathbf{q}$ is a row of $\mathbf{Q}$ (see p. 44 in [8]). However, the value of this correlation depends on the particular generalized inverse that is used to solve the MME (often implicitly) and is, thus, not uniquely defined (see p. 44 in [8]). A practical solution is to calculate the reliabilities from a model without UPG, but results in reliabilities to be overestimated because it ignores uncertainty due to the estimation of UPG [9].

To account for an increase in inbreeding and relationships, VanRaden [10] considered UPG as random effects by assigning them an inbreeding coefficient equal to the average inbreeding of known parents from the same generation. In crosses of populations, Lo et al. [11] modeled the covariances across animals by considering the heterogeneity of the base populations (i.e., different allele frequencies). Their model requires knowledge of the genetic variance of each base population and the segregation variance for each cross, which can differ for each trait. Garcia-Cortés and Toro [12] presented an equivalent model.

In a genomic context and with access to genotypes, Legarra et al. [13] introduced the concept of metafounders, which are a generalization of genetic groups that account for inbreeding and segregation variances (assumed the same for all traits) by introducing relatedness between base populations—the more different the base populations are, the larger is the genetic segregation variance. However, a base population from which all relationships are computed must be defined when considering different populations and crosses. For the case of two populations, Lo et al. [11] proposed using the F2 cross as a “reference group”. Mostly for convenience reasons (compatibility with genomic relationships), metafounders have a conceptual base population that has maximum heterozygosity. This lacks a clear genetic interpretation, as it differs from the base population of a pedigree without metafounders.

Using random UPG or metafounders leads to a model where all components of ${\mathbf{u}}^{*}$ are random and, thus, $re{l}_{i}=1-\frac{\text{Var}\left({\widehat{u}}_{i}-{u}_{i}\right)}{\text{Var}\left({u}_{i}\right)}$ holds. However, in preliminary tests we observed that the computed reliabilities for a model with metafounders were biased (too low), for instance, for proven dairy sires. Thus, we hypothesize that the reliabilities obtained from the inverse of the coefficient matrix when using metafounders do not refer to a meaningful breeding value in the usual quantitative genetics sense because they do not refer to a single unrelated base population. In fact, it is not obvious what the genetic base is when using random UPG or metafounders. Instead, we argue that $re{l}_{i}=1-\frac{\text{Var}\left({\widehat{u}}_{i}-{u}_{i}\right)}{\text{Var}\left({u}_{i}\right)}$ does represent the squared correlation between the true and estimated breeding values, but across two sampling processes. In the case of metafounders, these two sampling processes are: (i) from the conceptual base population of metafounders (one with maximum heterozygosity) to the metafounders, and (ii) from each metafounder to the individual. In the case of random UPG, there is a sampling from 0 (average UPG effect value a priori) to UPG values and then individual Mendelian samplings for each individual, which sum to UPG fractions, yield the final breeding value. Thus, in order to compute meaningful reliabilities of EBV we must define a proper base (or reference, as in Lo et al. [11]) population, relative to which the breeding values are expressed [14]. Thus, the objectives of this study were to develop theory and computational methods to obtain meaningful reliabilities for EBV for models with metafounders. The method is illustrated with data from the Lacaune dairy breed under pedigree-based and genomic-based models.

## Methods

### Theory

Consider the following linear mixed model:

1 $$\mathbf{y}=\mathbf{X}{\varvec{\upbeta}}+\mathbf{Z}\mathbf{u}+\mathbf{e},$$

where $\mathbf{y}$ is the vector of phenotypes, ${\varvec{\upbeta}}$ is the vector of fixed effects, $\mathbf{u}$ is the vector of breeding values, $\mathbf{e}$ is the vector of errors, and $\mathbf{X}$ and $\mathbf{Z}$ are incidence matrices. As usual, it is assumed that:

$$\text{E}\left[\mathbf{y}\right]=\mathbf{X}{\varvec{\upbeta}},$$

2 $$\text{and}\;\text{Var}\left(\begin{array}{c}\mathbf{u}\\ \mathbf{e}\end{array}\right)=\left(\begin{array}{cc}\mathbf{K}{\upsigma }_{u}^{2}& {\mathbf{0}} \\ {\mathbf{0}} & \mathbf{R}\end{array}\right).$$

The coefficient matrix of the MME for the model in Eq. (1) is:

3 $$\mathbf{C}=\left(\begin{array}{cc}{\mathbf{C}}_{11}& {\mathbf{C}}_{12}\\ {\mathbf{C}}_{21}& {\mathbf{C}}_{22}\end{array}\right)=\left(\begin{array}{cc}{\mathbf{X}}^{\mathbf{\prime}}{\mathbf{R}}^{-1}\mathbf{X}& {\mathbf{X}}^{\mathbf{\prime}}{\mathbf{R}}^{-1}\mathbf{Z}\\ {\mathbf{Z}}^{\mathbf{\prime}}{\mathbf{R}}^{-1}\mathbf{X}& {\mathbf{Z}}^{\mathbf{\prime}}{\mathbf{R}}^{-1}\mathbf{Z}+{\upsigma }_{u}^{-2}{\mathbf{K}}^{-1}\end{array}\right).$$

If the inverse of $\mathbf{C}$ is $${\mathbf{C}}^{-1}=\left(\begin{array}{cc}{\mathbf{C}}^{11}& {\mathbf{C}}^{12}\\ {\mathbf{C}}^{21}& {\mathbf{C}}^{22}\end{array}\right)$$, the reliability for the $i$th animal is calculated as [7]:

4 $$re{l}_{i}=1-\frac{{\mathbf{C}}_{ii}^{22}}{{\mathbf{K}}_{ii}{\sigma }_{u}^{2}}=1-\frac{\text{Var}\left({\widehat{u}}_{i}-{u}_{i}\right)}{\text{Var}\left({u}_{i}\right)}.$$

In “regular” animal models, $\mathbf{K}=\mathbf{A}$, where $\mathbf{A}$ is the numerator relationship matrix. Now, we introduce a distinction by using metafounders. We keep ${u}_{i}$ for the breeding value of individual $i$ estimated in the “regular” animal model, and ${u}_{i}^{*}$ for the breeding value estimated with metafounders. When using metafounders [13], ${\mathbf{K}}^{-1}$ in Eq. (3) is replaced by either ${\mathbf{H}}_{{\varvec{\Gamma}}}^{-1}$ or ${\mathbf{A}}_{{\varvec{\Gamma}}}^{-1}$ if single-step genomic best linear unbiased prediction (ssGBLUP) [13, 15, 16] or pedigree-based BLUP is used, respectively. For the structure of ${\mathbf{H}}_{{\varvec{\Gamma}}}^{-1}$ and ${\mathbf{A}}_{{\varvec{\Gamma}}}^{-1}$, see [13]. In addition, ${\sigma }_{u}^{2}$ in Eqs. (3) and (4) is substituted by ${\sigma }_{u{\text{-}}related}^{2}$, which is the genetic variance accounting for relatedness in the base population [13, 17].

Using metafounder models, the reliability calculated following Eq. (4) (i.e., $1-\frac{\text{Var}\left({\widehat{u}}_{i}^{*}-{u}_{i}^{*}\right)}{\text{Var}\left({u}_{i}^{*}\right)}$) will be referred to as ${rel}_{i}^{*}$. Initial empirical evidence (examples will be shown later) and analytical proofs (see Appendix) showed that calculating reliabilities with metafounders based on Eq. (4) leads to reliability values that are lower than expected—for instance, 0.60 for a proven bull with 100 progenies with records in a pedigree-BLUP evaluation with ${h}^{2}=0.25$. This is because Eq. (4) refers to a base population of maximum heterozygosity [13] and with an expected value equal to zero, which has no meaning for breeding purposes. Metafounders are conceptually drawn from this base population with covariance matrix ${\varvec{\Gamma}}{\sigma }_{u{\text{-}}related}^{2}$ [13], and breeding values of individual animals are subsequently drawn through Mendelian sampling. Although this conceptual base population has no meaningful genetic interpretation, metafounders do. For example, they represent founders of the pedigree [18], founders of pure breeds [19], or unknown parents of animals born in given time periods [20]. In order to refer reliabilities to a base population [14], we propose to define the reliabilities as a contrast to one of these populations, i.e., contrasts to a reference metafounder.

#### Reliability as a contrast

Thus, we define the reliability $re{l}_{{c}_{i}}$ of the contrast of “the EBV of animal $i$ minus the estimated effect of the metafounder $mf$”, in the usual manner as $re{l}_{{c}_{i}}={r}^{2}\left({\widehat{u}}_{i}^{*}-{\widehat{u}}_{mf}^{*},{u}_{i}^{*}-{u}_{mf}^{*}\right)$, with the following analytical expression:

$$re{l}_{{c}_{i}}=1-\frac{\text{Var}\left(\left({\widehat{u}}_{i}^{*}-{u}_{i}^{*}\right)-\left({\widehat{u}}_{mf}^{*}-{u}_{mf}^{*}\right)\right)}{\text{Var}\left({u}_{i}^{*}-{u}_{mf}^{*}\right)}$$

5 $$=1-\frac{{\mathbf{C}}_{{\Gamma }_{ii}}^{22}+{\mathbf{C}}_{{\Gamma }_{mf,mf}}^{22}-2{\mathbf{C}}_{{\Gamma }_{i,mf}}^{22}}{\left({\mathbf{V}}_{{\Gamma }_{ii}}+{\mathbf{V}}_{{\Gamma }_{mf,mf}}-2{\mathbf{V}}_{{\Gamma }_{i,mf}}\right){\sigma }_{\text{u-related}}^{2}},$$

where $\left({\widehat{u}}_{mf}^{*}\right) {u}_{mf}^{*}$ is the (estimated) breeding value of the chosen reference metafounder. In Eq. (5), ${\mathbf{V}}_{{\Gamma }_{ii}}$ refers to either ${\mathbf{A}}_{{\Gamma }_{ii}}$ or ${\mathbf{H}}_{{\Gamma }_{ii}}$, whereas ${\mathbf{C}}_{{\Gamma }_{j,k}}^{22}$ refers to the $j$ $k$ element of the corresponding block of the inverse of $\mathbf{C}$ calculated by replacing ${\upsigma }_{u}^{-2}{\mathbf{V}}^{-1}$ by ${\sigma }_{\text{u-related}}^{-2}{\mathbf{A}}_{{\varvec{\Gamma}}}^{-1}$ or ${\sigma }_{\text{u-related}}^{-2}{\mathbf{H}}_{{\varvec{\Gamma}}}^{-1}$. Note that Eq. (5) can be also used to calculate the reliability of the contrast of the estimated effect of two metafounders. Hence, it is possible to obtain a statistic to evaluate the precision of the estimate of a metafounder’s effect, something which is not easy to do for fixed-effect UPG.

#### Comparison to “regular” animal model reliabilities

Let $re{l}_{i}=1-\frac{\text{Var}\left({\widehat{u}}_{i}-{u}_{i}\right)}{{A}_{\text{ii}}{\sigma }_{u}^{2}}$ be the “regular” animal model reliability without UPG or metafounders. In Appendix (Eq. 20), we show that for the case of a single metafounder and under mild assumptions, $re{l}_{i}=re{l}_{{c}_{i}}$. This shows that our proposal for $re{l}_{{c}_{i}}$ is a generalization of standard animal breeding theory [7]. The assumption for the proof in the Appendix to hold is that the product of the incidence matrix for the random effects ($\mathbf{Z})$ times a vector of 1s ($\mathbf{1}$) must belong to the column space of the incidence matrix $\mathbf{X}$ for the fixed effects ($\mathbf{Z1}\in \mathcal{C}\left(\mathbf{X}\right)$, the column space of $\mathbf{X}$). From an interpretational point of view, this assumption implies that an overall mean is fitted implicitly or explicitly in the model ($\mathbf{1}\in \mathcal{C}\left(\mathbf{X}\right)$) and that the sum of each row of the incidence matrix $\mathbf{Z}$ is constant ($\mathbf{Z1}\propto \mathbf{1}$). This assumption holds for a wide variety of models, including sire, animal (with and without maternal effects), and multi-trait models. For more complex models, such as competition models [21] and models with indirect genetic effects with non-constant group sizes [22], the assumption seems to hold if the models are sensibly specified.

We prove in the Appendix that the expression in Eq. (4) ${rel}_{i}^{*}=1-\frac{\text{Var}\left({\widehat{u}}_{i}^{*}-{u}_{i}^{*}\right)}{Var\left({u}_{i}^{*}\right)}$ gives a systematically lower value of reliability than the regular $re{l}_{i}$; in fact, ${rel}_{i}^{*}=\frac{\left(2-\upgamma \right){\mathbf{A}}_{ii}}{\left(2-\upgamma \right){\mathbf{A}}_{ii}+2\upgamma } {rel}_{i}$ (see Eq. (19) in Appendix). This is because $\gamma$ is twice the average inbreeding coefficient of a population; hence, it ranges from 0 to 2 and, therefore, the scalar $\frac{\left(2-\gamma \right){A}_{ii}}{\left(2-\gamma \right){A}_{ii}+2\gamma }$ always ranges from 0 to 1. For instance, when ${A}_{ii}=1$ (no inbreeding) this gives ${rel}_{i}^{*}=\frac{2-\gamma }{2+\gamma } re{l}_{i}$, and for a typical value of $\gamma$, say $0.6$, this gives ${rel}_{i}^{*}=0.54re{l}_{i}$.

#### Reliabilities of metafounders

A point that is overlooked in genetic evaluations with UPG is the precision of the estimation of UPG values [9]. In principle, it is possible to compute the standard error of the contrasts of the estimates of two UPG from the elements of the inverse of the MME. However, to our knowledge, this is usually not done. In our study, the reliability of a contrast (Eq. 5) can also be applied to metafounders, which are treated like any other animals. Thus, it is possible to obtain reliabilities of contrasts of metafounders on the same 0 to 1 scale as individuals. So, the reliability of metafounder 2 compared to metafounder 1 also uses Eq. (5). Contrary to contrasts of UPG, the reliabilities of contrasts of metafounders do not need special computational treatment.

### Computing strategies

For small datasets, the MME can be solved by inversion of $\mathbf{C}$. Therefore, ${\mathbf{C}}_{{\Gamma }_{ii}}^{22}$, ${\mathbf{C}}_{{\Gamma }_{mf,mf}}^{22}$, and ${\mathbf{C}}_{{\Gamma }_{i,mf}}^{22}$ of Eq. (5) can be obtained from ${\mathbf{C}}^{-1}$. Also, ${\mathbf{K}}_{\Gamma }$ can be explicitly created and used to obtain the elements for the denominator of Eq. (5).

For large datasets without genomic information, ${\mathbf{C}}_{{\Gamma }_{ii}}^{22}$ can be retrieved from a sparse inverse of $\mathbf{C}$, or approximated using a reliability approximation method, e.g. [23, 24], or by a Gibbs sampler. The elements ${\mathbf{C}}_{{\Gamma }_{mf,mf}}^{22}$ and ${\mathbf{C}}_{{\Gamma }_{i,mf}}^{22}$ are obtained in $\mathbf{x}$ by solving the system $\mathbf{C}\mathbf{x}={\mathbf{e}}_{mf}$, where ${\mathbf{e}}_{mf}$ is a vector of 0s except for a 1 in the position of the reference metafounder. Note that the system needs to be solved only once, for the reference metafounder. The diagonal elements of ${\mathbf{A}}_{\Gamma }$ (${\mathbf{A}}_{{\Gamma }_{ii}}$ and ${\mathbf{A}}_{{\Gamma }_{mf,mf}}$) are calculated before setting up the MME, using either a modified version of the Meuwissen and Luo [25] algorithm [13] or recursion. The elements ${\mathbf{A}}_{{\Gamma }_{mf,i}}$ are obtained by applying the method of Colleau [22], modified to account for metafounders.

For large datasets with genomic information, ${\mathbf{C}}_{{\Gamma }_{ii}}^{22}$ can still be obtained from a sparse inverse of $\mathbf{C}$ if the number of genotyped animals is small. Otherwise, ${\mathbf{C}}_{{\Gamma }_{ii}}^{22}$ can be approximated by a Gibbs sampler or by reliability approximation methods that account for genomic information, e.g. [26–28]. The calculation of ${\mathbf{C}}_{{\Gamma }_{mf,mf}}^{22}$ and ${\mathbf{C}}_{{\Gamma }_{i,mf}}^{22}$ is the same as with pedigree only. The diagonal elements of ${\mathbf{H}}_{\Gamma }$ can be calculated using the methods of Legarra et al. [29]. Finally, the elements ${\mathbf{H}}_{{\Gamma }_{i,mf}}$ can be obtained in $\mathbf{x}$ by solving the system ${\mathbf{H}}_{{\varvec{\Gamma}}}^{-1}\mathbf{x}={\mathbf{e}}_{mf}$. A more efficient way would be to solve separate systems for genotyped and non-genotyped animals. For genotyped animals, it is necessary to calculate $\mathbf{x}=\mathbf{G}{\mathbf{A}}_{{\Gamma }_{22}}^{-1}{\mathbf{A}}_{{\Gamma }_{21}}{\mathbf{e}}_{mf}$, where the subscripts $1$ and 2 refer to the non-genotyped and genotyped animals, respectively. First, the product $\mathbf{w}={\mathbf{A}}_{{\Gamma }_{22}}^{-1}{\mathbf{A}}_{{\Gamma }_{21}}{\mathbf{e}}_{mf}$ can be efficiently calculated using the method of Fernando et al. [30], as the solution to ${\mathbf{A}}_{{\varvec{\Gamma}}}^{22}\mathbf{w}={-\mathbf{A}}_{\varvec{\Gamma }}^{21}{\mathbf{e}}_{mf}$. Then, values of $\mathbf{x}$ for genotyped animals are obtained from $\mathbf{x}=\mathbf{G}\mathbf{w}$. For non-genotyped animals, it is necessary to calculate ${\mathbf{x}=\mathbf{A}}_{{\Gamma }_{11}}{\mathbf{e}}_{mf}+{\mathbf{A}}_{{\Gamma }_{12}}{\mathbf{A}}_{{\Gamma }_{22}}^{-1}\left(\mathbf{G}-{\mathbf{A}}_{{\Gamma }_{22}}\right){\mathbf{A}}_{{\Gamma }_{22}}^{-1}{\mathbf{A}}_{{\Gamma }_{21}}{\mathbf{e}}_{mf}$. The first term is calculated using the method of Colleau [31], whereas the second uses multiplications from right to left. For more details on these methods, we refer to Colleau et al. [32].

The above methods to implement Eq. (5) have been programmed in the BLUPF90+ [33] software and may be invoked by adding the following options to the parameter file:

OPTION store_accuracy n

OPTION store_pec_mf arg

where *n* refers to the number of the animal effect and *arg* defines which metafounder is used as a contrast (first, last, user nmf). The default uses the first (first) metafounder, but any metafounder (nmf) can be defined as a contrast through the argument user nmf.

### Materials

The proposed method was tested on a sheep dataset from the Lacaune breed. The number of animals in the pedigree was 1,961,687 (primarily females), from which 1,791,268 had phenotypes for milk yield. In total, 29,138 rams were genotyped with a 50K single nucleotide polymorphism (SNP) chip. The pedigree records started in 1970 and the unknown parents of sheep until 1978 constituted the first metafounder. Then, metafounders were created every 2 years. Although pedigree completeness was above 90% and all males born after 1978 had both parents known, there are females with unknown sire or unknown sire and dam. Table 1 shows the number of animals with records assigned to each metafounder, which ranged from 2000 to 100,000.

**Table 1:** Definition of metafounders according to a 2-year interval span and number of animals with records per metafounder

Metafounder code	Metafounder year	Number of animals with records
1	< 1978	102,699
2	1978	17,052
3	1980	15,278
4	1982	14,604
5	1984	14,150
6	1986	13,806
7	1988	12,580
8	1990	11,357
9	1992	10,529
10	1994	10,695
11	1996	10,154
12	1998	12,908
13	2000	10,652
14	2002	8760
15	2004	6808
16	2006	6291
17	2008	7299
18	2010	6159
19	2012	5792
20	2014	5414
21	2016	5431
22	2018	4834
23	2020	1678

### Analysis

Two models (BLUP and ssGBLUP) with three versions of reliability scenarios were used to explore the properties of regular reliability and the reliability of the contrast between an individual’s EBV and the metafounder’s effect. For BLUP, the first scenario did not include UPG or metafounders. The reliabilities calculated from this model $Re{l}_{i}=1-\frac{\text{Var}\left({\widehat{u}}_{i}-{u}_{i}\right)}{{\mathbf{K}}_{ii}{\sigma }_{u}^{2}}$ will be referred to as *RelNoMF* for BLUP and serve as a reference of properly defined and accurate reliabilities, as most animals have complete pedigree. For ssGBLUP, this scenario was not considered, as genomic reliabilities depend strongly on the genotype coding [14, 34]. The second scenario (used for BLUP and ssGBLUP) included metafounders but without any correction for the reference population. Thus, the reliability was calculated following Eq. (4): $Re{l}_{i}^{*}=1-\frac{\text{Var}\left({\widehat{u}}_{i}^{*}-{u}_{i}^{*}\right)}{{\mathbf{K}}_{{\Gamma }_{i,i}}{\sigma }_{u{\text{-}}related}^{2}}$. For this method, the reliability calculated from the inverse of the MME coefficient matrix refers to a genetic base population with maximum heterozygosity, which will be referred to as *RelMFnc* for BLUP and *ssRelMFnc* for ssGBLUP. Here, we compute reliabilities with sparse inversion for ssGBLUP because the use of metafounders with estimated ${\varvec{\Gamma}}$ puts $\mathbf{G}$ and $\mathbf{A}$ on the “same scale” [13, 17], leading to meaningful reliabilities. The third scenario used the same model with metafounders but reliabilities were defined as a contrast to a reference metafounder, as proposed in this study in Eq. (5): $Re{l}_{{c}_{i}}=1-\frac{\text{Var}\left(\left({\widehat{u}}_{i}^{*}-{u}_{i}^{*}\right)-\left({\widehat{u}}_{mf}-{u}_{mf}\right)\right)}{Var\left({u}_{i}^{*}-{u}_{mf}\right)}$ and will be referred to as *RelMFc* and *ssRelMFc* for BLUP and ssGBLUP, respectively. The reference metafounder was the first one, which happens to represent the “oldest” Lacaune population.

For all the scenarios with metafounders, their relationship matrix $({\varvec{\Gamma}})$ was calculated using a modification of the method of Macedo et al. [20] as:

6 $${\varvec{\Gamma}}=\mathbf{11}^{\mathbf{\prime}}{\widehat{\upgamma }}_{0}+2k\mathbf{T}{\mathbf{T}}^{\mathbf{\prime}}\Delta {F}_{\upgamma },$$

where ${\widehat{\upgamma }}_{0}$ is the estimate of the self-average relationship of the first metafounder $\left({\gamma }_{0}\right)$, $k$ is the time (in years) between consecutive metafounders, $\mathbf{T}$ is a strictly lower triangular matrix of 1s, and $\Delta {F}_{\upgamma }=\Delta F\left(1-0.5 {\upgamma }_{0}\right)$, where $\Delta F$ is the change in average inbreeding per year (assumed constant but this can be modified). For the dataset used in this study, $k=2$ and $\Delta F=6.442 \times {10}^{-4}$ per year as obtained by pedigree analyses. Parameter ${\gamma }_{0}$ was estimated as ${\widehat{\upgamma }}_{0}=2 {n}^{-1}{\sum }_{i=1}^{n}{\left(2 {\widehat{p}}_{i}-1\right)}^{2}$ [18], where $n$ is the number of genotyped markers and ${\widehat{p}}_{i}$ is the estimate of the minor allele frequency in the base population for the $i$th marker, which was calculated using the method of Gengler et al. [35], resulting in ${\widehat{\upgamma }}_{0}=0.46375$. Note that this technique to obtain ${\varvec{\Gamma}}$ is only valid in the context of a single breed and cannot be used when the population includes several breeds and crosses. For simplicity, we assumed a continuous decrease in heterozygosity, but this can be easily relaxed. All computations were done using BLUPF90+ [33].

Reliabilities obtained from each scenario were compared for all animals and by the following categories of animals: selection candidates and males and females with two, one, or no parents known. Selection candidates were defined as genotyped young males without progeny.

## Results

**Fig. 1:**
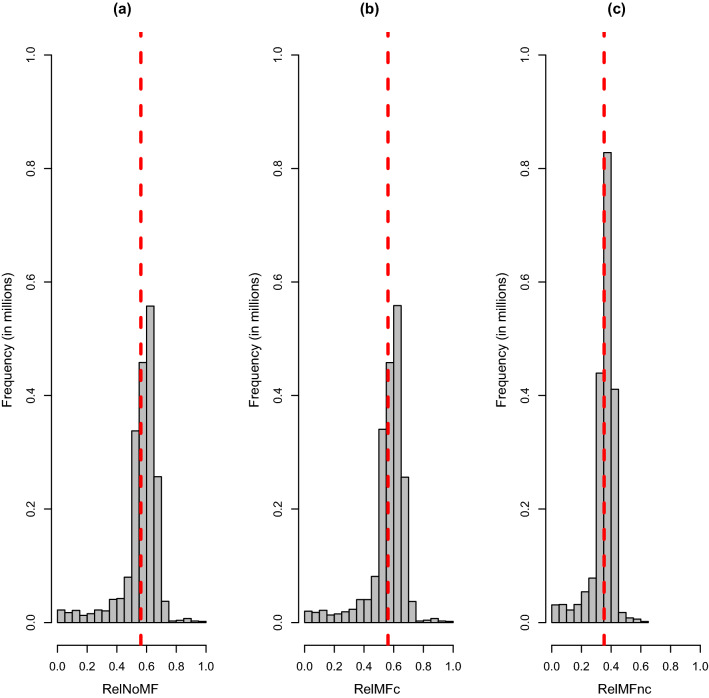
Histogram of the three reliabilities for all animals: **a** *RelNoMF*, **b** *RelMFc*, and **c** *RelMFnc*. The dashed red line denotes the average reliability for each scenario

**Fig. 2:**
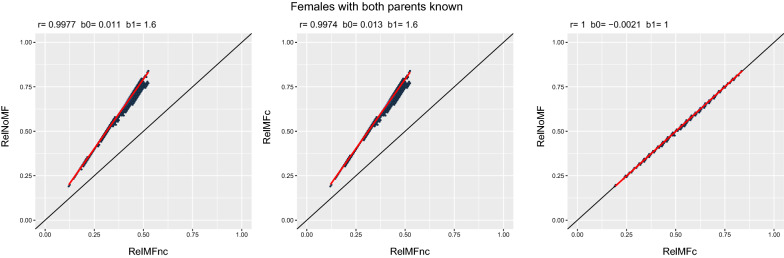
Scenario comparison for females with both parents known. The red line is the fitted regression

Figure 1 shows histograms for reliabilities for *RelNoMF*, *RelMFc*, and *RelMFnc*. The leftmost and larger peaks contain females, most having own records, whereas the rightmost and smaller peak consists of proven males. The average reliabilities of ~ 0.55 for females and ~ 0.90 for proven males in Fig. 1 agree with values from simple selection index theory, as the females have one to three records and proven males have a minimum of 30 daughters each, the heritability of the trait was 0.30 and the repeatability was 0.50. The values for *RelNoMF* and *RelMFc* align extremely well, as also shown in Fig. 2 (for females with both parents known). However, *RelMFnc* resulted in reliabilities that are too low both for males (~ 0.55) and females (~ 0.40), as expected based on the expression $Re{l}_{i}^{*}=\frac{\left(2-\upgamma \right){\mathbf{A}}_{ii}}{\left(2-\upgamma \right){\mathbf{A}}_{ii}+2\upgamma } {rel}_{i}$. *RelMFnc* also gave reliabilities that are too low because they refer to a conceptual base population with maximum heterozygosity.

**Fig. 3:**
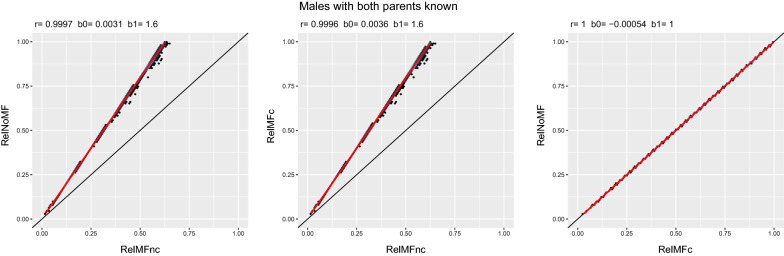
Scenario comparison for males with both parents known. The red line is the fitted regression

Figures 2 and 3 show the comparison between scenarios using BLUP for females and males, respectively. The leftmost plots in Figs. 2 and 3, i.e., the comparison between *RelNoMF* and *RelMFnc* evidence the inadequacy of Eq. (4) for calculating reliabilities with metafounders. The dots over the red dashed line indicate that the reliability is miscalculated because the addition of metafounders in *RelMFnc* should result in very similar reliabilities as *RelNoMF*, given that most individuals have both parents known. The comparison between *RelMFc* against *RelMFnc* in Figs. 2 and 3 demonstrate the underestimation of the reliabilities when using metafounders without the correction that is suggested in Eq. (5). Finally, the rightmost plots show how the calculation of the reliability as a contrast with a reference metafounder corrects the underestimation in *RelMFnc*.

**Fig. 4:**
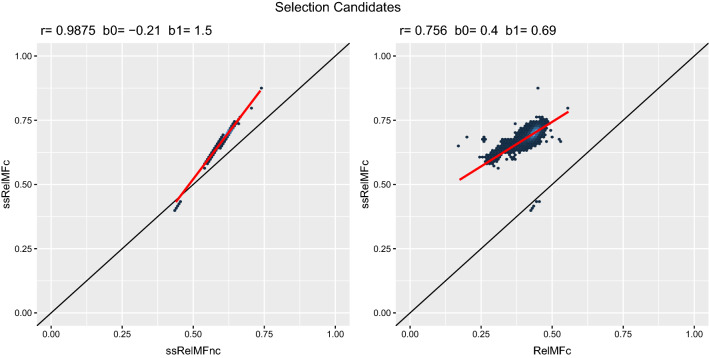
Comparison between *ssRelMFnc*, *ssRelMFc*, and *RelMFc* for selection candidates

For ssGBLUP, because the vast majority of individuals are not genotyped, results for non-genotyped animals were equal to those from Figs. 2 and 3. For genotyped selection candidates (males with genotype but no offspring yet), Fig. 4 shows the comparison between *ssRelMFnc* and *ssRelMFc*. Again, *ssRelMFnc* was systematically lower than *ssRelMFc*, with the mode for genotyped selection candidates approximately equal to 0.62 for *ssRelMFnc* and 0.70 for *ssRelMFc*. Given that *ssRelMFnc* systematically underestimated reliabilities, *ssRelMFc* provides accurate reliabilities*.* The comparison of *ssRelMFc* and *RelMFc* in Fig. 4 shows the gain in reliability by adding genomic information.

**Fig. 5:**
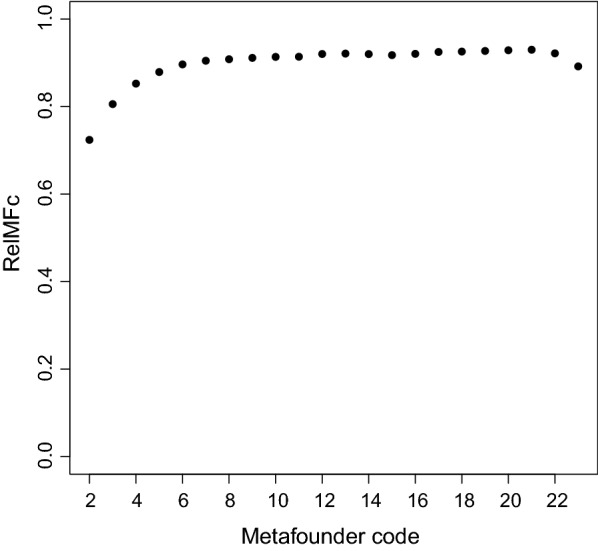
Reliability for selection candidates using the contrast between all the metafounders and the reference metafounder

Finally, Fig. 5 shows the reliabilities of the contrasts of each metafounder with the reference metafounder. Most reliabilities oscillated around 0.90. In particular, the reliabilities declined in the most recent years because there is less information. Given the large number of records per metafounder (Table 1), we expected higher reliabilities. This may illustrate that correct estimation of different base populations (metafounders or UPG) is difficult, probably due to confounding of metafounders with environmental effects (flock-year) and poor genetic connections between metafounders (which, at best, are based through many common offspring of metafounders).

## Discussion

We have not been able to find a discussion in the scientific literature on the definition of reliabilities for models with fixed UPG. Presumably, this is mainly because UPG are used in large datasets and reliabilities for EBV are approximated while ignoring UPG [23, 24, 36]. Another reason might be that reliabilities are not uniquely defined because the inverse of the additive relationship matrix is non-full rank [8]. Da et al. [37] presented methods to calculate prediction error variances for models with fixed UPG. Although these prediction error variances can be used in REML procedures, they cannot be used to obtain reliabilities because these prediction error variances and their derived reliabilities depend on the choice of the generalized inverse that is used to solve the mixed model equations. Hickey et al. [38] proposed a sampling-based method to calculate the reliability of EBV by simulating the true breeding value using random UPG. However, in this method, UPG effects are either drawn as random and estimated as random (in which case the associated variance component is unclear and the reliabilities refer to an undefined population), or UPG effects are drawn as fixed and estimated as fixed (in which case they suffer the same problem of lack of uniqueness). In our opinion, metafounders is a structure that is better defined from a quantitative genetics point of view, as relationships between metafounders are functions of heterozygosity at markers [18].

Another point is how to define a base point in genetic evaluations [14]. The metafounder approach uses a conceptual, non-existing population that has maximum heterozygosity, which leads to reliabilities that are too low, i.e. in some way, the maximum heterozygosity population is too distant from the actual animals—for the difference to be correctly estimated. In animal breeding practice, EBV are often referred to the average of a group of recent animals, such as “purebred females born in a given year”. This suggests that reliabilities should be defined as contrasts from the same average, which is conceptually feasible but not practical as it would imply manipulating thousands of prediction error variances and covariances. Thus, the breeder must choose a metafounder to provide a base for the contrast. In purebreds, a meaningful choice is an “old” population (representing the oldest founders of the breed), but it could also be the metafounder that has the most unknown parents assigned to it. In crossbreds, a natural choice is one of the parental breeds (or the oldest population within the breed), or even different breeds, which would result in different reliabilities for contrasts from different breeds. Our proposal of using metafounders within breeds assumes that differences in variances per breed and segregation variances are correctly defined by the matrix ${\varvec{\Gamma}}$.

Using genomic information added another layer of complexity to the interpretation of reliabilities calculated from the inverse of the MME. Stranden and Christensen [34] showed that different allele coding results in different reliabilities, although the EBV remain the same. Tier et al. [14] proposed a method to obtain prediction error variances that are independent of allele coding for GBLUP. Stranden et al. [39] proposed a method to fit the so-called J-factors and genetic groups, similar to metafounders, but as fixed instead of random effects. One of the drawbacks of their method is that the reliability is undefined. Our proposal of using metafounders together with contrasts overcomes these issues since the arbitrariness of allele frequencies is overcome by fixing them to 0.5 and the base population to which the reliabilities refer is explicitly addressed. Thus, our method adequately reports reliabilities of EBV from ssGBLUP models with different base populations.

An issue with the use of metafounders is the variance components that should be used for the covariance structure because they have no clear genetic interpretation without the associated ${\varvec{\Gamma}}$. For a single metafounder, the variance components associated with the metafounder and the genetic (co)variance of pedigree founders are proportional by $\left(1-\frac{\gamma }{2}\right)$ [13]. For several metafounders, the proportionality constant is $\left(1+\frac{\overline{diag\left({\mathbf{\Gamma}}\right)}}{2}-\overline{{\varvec{\Gamma}} }\right)$ [13]. However, this assumes a mixture in equal proportions of all individuals’ origins, which is not necessarily valid. Thus, future research could focus on which covariance matrix should be used when many metafounders are fitted in a model. In our Lacaune example, the scaling factor $1/\left(1+\frac{\overline{diag\left({\mathbf{\Gamma}}\right)}}{2}-\overline{{\varvec{\Gamma}} }\right)$ resulted in a value of 0.7654, which is very close to $1/\left(1-\frac{{\Gamma }_{\left({1,1}\right)}}{2}\right)$ (where ${\Gamma }_{\left({1,1}\right)}$ = 0.4638) because the increase in coancestry over time is very small compared to the initial heterozygosity of the breed. This may be true when using metafounders to model missing pedigrees within a breed.

However, in breed crosses, it is not obvious how to obtain variance components with metafounders from “routine” evaluations. The current modeling of breed crosses does not consider segregation variances, so a model with metafounders is just different. However, Poulsen et al. [40] showed by simulation that there is very good agreement between simulated segregation variances and their expected values based on ${\varvec{\Gamma}}$ multiplied by a single variance component ${\sigma }_{u{\text{-}}related}^{2}$. Nevertheless, more work is needed in this regard. Alternatively, one could simply estimate ${\sigma }_{u{\text{-}}related}^{2}$ and, if needed, express genetic variances on the usual “unrelated” scale, combining it with ${\varvec{\Gamma}}$ [41].

As mentioned before, Eq. (5) allows the reliability of the estimates of genetic groups or metafounders with respect to a reference group to be computed. Reliabilities of the effects of genetic groups are usually not calculated in genetic evaluations. The proposed Eq. (5) could help identify non-reliable groups, which can be a sign of non-optimal assignment of genetic groups. For example, if 2 consecutive metafounders defined by year of birth have a low reliability, combining them would allow more accurate modeling of genetic groups.

## Conclusions

To date, there was no expression for the reliabilities with different base populations. Reliabilities that are calculated directly from prediction error variances obtained from the inverse of MME in models with metafounders underestimate the reliability of EBV because they refer to a conceptual base population that has maximum heterozygosity. We propose to calculate reliability as a contrast to a chosen metafounder. This leads to expressions for reliability that are mathematically identical to those for a single population and using pedigree-BLUP, and our empirical results show that they are adequate when there are several base populations. Given that computations are not difficult, we propose that reliabilities as a contrast to a reference metafounder should be used in routine evaluations, when different base populations are fitted into the model, both for BLUP and ssGBLUP.

## Data Availability

The data used in this work is owned by the organizations responsible for the Lacaune breeding program (Upra Lacaune, Ovitest, Confederation Générale de Roquefort), to whom it may be requested.

## Prediction error variances and reliabilities with a single metafounder

This Appendix shows that, under some assumptions, when there is a single metafounder that represents pedigree founders, the direct reliability of the breeding value has a lower numerical value than for the regular animal model with no metafounder. We also prove that the reliability of the contrast with a single metafounder is mathematically identical to the regular animal model with no metafounder.

### A mixed model property

Let a mixed model be:

$$\mathbf{y}=\mathbf{X}{\varvec{\upbeta}}+\mathbf{Z}\mathbf{u}+\mathbf{e},$$

$$\text{E}\left[\mathbf{y}\right]=\mathbf{X}{\varvec{\upbeta}},$$

7 $$\text{Var}\left(\begin{array}{c}\mathbf{u}\\ \mathbf{e}\end{array}\right)=\left(\begin{array}{cc}\mathbf{K}{\upsigma }_{u}^{2}& \mathbf{0} \\ \mathbf{0} & \mathbf{R}\end{array}\right),$$

with the mixed model equations:

8 $$\mathbf{C}=\left(\begin{array}{cc}{\mathbf{C}}_{11}& {\mathbf{C}}_{12}\\ {\mathbf{C}}_{21}& {\mathbf{C}}_{22}\end{array}\right)=\left(\begin{array}{cc}{\mathbf{X}}^{\mathbf{\prime}}{\mathbf{R}}^{-1}\mathbf{X}& {\mathbf{X}}^{\mathbf{\prime}}{\mathbf{R}}^{-1}\mathbf{Z}\\ {\mathbf{Z}}^{\mathbf{\prime}}{\mathbf{R}}^{-1}\mathbf{X}& {\mathbf{Z}}^{\mathbf{\prime}}{\mathbf{R}}^{-1}\mathbf{Z}+{\upsigma }_{u}^{-2}{\mathbf{K}}^{-1}\end{array}\right),$$

and inverse $${\mathbf{C}}^{-1}=\left(\begin{array}{cc}{\mathbf{C}}^{11}& {\mathbf{C}}^{12}\\ {\mathbf{C}}^{21}& {\mathbf{C}}^{22}\end{array}\right)$$. Let $\mathbf{Z1}\in \mathcal{C}\left(\mathbf{X}\right)$ (the column space of $\mathbf{X}$), then ${\mathbf{C}}^{22}{\mathbf{K}}^{-1}{\mathbf{1}}={\mathbf{1}}{\upsigma }_{u}^{2}$. That is, ${\upsigma }_{u}^{2}$ is an eigenvalue of ${\mathbf{C}}^{22}{\mathbf{K}}^{-1}$, and its associated eigenvector is $\mathbf{1}$.

#### *Proof*

By partitioned matrix inverse rules [42], ${\mathbf{C}}^{22}={\left({\upsigma }_{u}^{-2}{\mathbf{K}}^{-1}+{\mathbf{Z}}^{\mathbf{\prime}}{\mathbf{R}}^{-1}\left(\mathbf{I}-{\mathbf{P}}_{\mathbf{X}}\right)\mathbf{Z}\right)}^{-1}$, where ${\mathbf{P}}_{\mathbf{X}}={\mathbf{X}\left({\mathbf{X}}^{\mathbf{\prime}}{\mathbf{R}}^{-1}\mathbf{X}\right)}^{-1}{\mathbf{X}}^{\mathbf{\prime}}{\mathbf{R}}^{-1}$ is an oblique projector operator onto the column space of $\mathbf{X}$. Then,

9 $$\mathbf{K}{{\mathbf{C}}^{22}}^{-1}{\mathbf{1}}=\mathbf{K}\left({\upsigma }_{u}^{-2}{\mathbf{K}}^{-1}{\mathbf{1}}+{\mathbf{Z}}^{\mathbf{\prime}}{\mathbf{R}}^{-1}\left(\mathbf{I}-{\mathbf{P}}_{\mathbf{X}}\right)\mathbf{Z1}\right)=\mathbf{K}\left({\upsigma }_{u}^{-2}{\mathbf{K}}^{-1}{\mathbf{1}}+{\mathbf{Z}}^{\mathbf{\prime}}{\mathbf{R}}^{-1}\left(\mathbf{Z1}-{\mathbf{P}}_{\mathbf{X}}\mathbf{Z1}\right)\right)=\mathbf{K}\left({\upsigma }_{u}^{-2}{\mathbf{K}}^{-1}{\mathbf{1}}+{\mathbf{Z}}^{\mathbf{\prime}}{\mathbf{R}}^{-1}\left(\mathbf{Z1}-\mathbf{Z1}\right)\right)={\upsigma }_{u}^{-2}\mathbf{K}{\mathbf{K}}^{-1}{\mathbf{1}}={\upsigma }_{u}^{-2}{\mathbf{1}}.$$

Since ${\upsigma }_{u}^{-2}$ is an eigenvalue of $\mathbf{K}{{\mathbf{C}}^{22}}^{-1}$ and $\mathbf{K}{{\mathbf{C}}^{22}}^{-1}$ is non-singular, then ${\upsigma }_{u}^{2}$ is an eigenvalue of ${\mathbf{C}}^{22}{\mathbf{K}}^{-1}$, and its associated eigenvector is $\mathbf{1}$.

### Reliability for models with one metafounder

When one metafounder is included in an animal model, the assumed linear mixed model is:

$${\mathbf{y}}=\mathbf{X}{\varvec{\upbeta}}+\left[\begin{array}{cc}{\mathbf{0}}& \mathbf{Z}\end{array}\right]{{\mathbf{u}}}^{*}+{\mathbf{e}},$$

$$\text{E}\left[{\mathbf{y}}\right]=\mathbf{X}{\varvec{\upbeta}},$$

10 $$\text{Var}\left(\begin{array}{c}{{\mathbf{u}}}^{\mathbf{*}}\\ {\mathbf{e}}\end{array}\right)=\left(\begin{array}{cc}{\mathbf{A}}_{\upgamma }{\upsigma }_{u{\text{-}}related}^{2}& {\mathbf{0}} \\ {\mathbf{0}} & \mathbf{R}\end{array}\right)=\left(\begin{array}{cc}{\mathbf{A}}_{\upgamma }w{\sigma }_{u}^{2}& {\mathbf{0}}\\ {\mathbf{0}}& \mathbf{R}\end{array}\right),$$

given that $$w={\left(1-\frac{\upgamma }{2}\right)}^{-1}$$,

11 $$\text{and}\;{\mathbf{A}}_{\upgamma }w=\left(\begin{array}{cc}\upgamma & \upgamma {\mathbf{1}}{^{\mathbf{\prime}}}\\\upgamma \mathbf{1} & \mathbf{A}\left(1-\frac{\upgamma }{2}\right)+\upgamma {\mathbf{11}}{^{\mathbf{\prime}}}\end{array}\right){\left(1-\frac{\upgamma }{2}\right)}^{-1},$$

where $\upgamma$ is a scalar. For the genetic interpretation of $\upgamma$, we refer the reader to Christensen [17] and Legarra et al. [13]. Using partitioned matrix inverse rules, it can be shown that $${\mathbf{A}}_{\upgamma }^{-1}{\left(w\right)}^{-1}=\left(\begin{array}{cc}{\upgamma }^{-1}\left(1-\frac{\upgamma }{2}\right)+{\mathbf{1}}^{\mathbf{\prime}}{\mathbf{A}}^{-1}\mathbf{1} & -{\mathbf{1}}^{\mathbf{\prime}}{\mathbf{A}}^{-1}\\ -{\mathbf{A}}^{-1}\mathbf{1}& {\mathbf{A}}^{-1}\end{array}\right).$$

The coefficient matrix of mixed model equations is:

$$\mathbf{M}=\left(\begin{array}{ccc}{\mathbf{M}}_{11}& {\mathbf{M}}_{12}& {\mathbf{M}}_{13}\\ {\mathbf{M}}_{21}& {\mathbf{M}}_{22}& {\mathbf{M}}_{23}\\ {\mathbf{M}}_{31}& {\mathbf{M}}_{32}& {\mathbf{M}}_{33}\end{array}\right)$$

12 $$=\left(\begin{array}{ccc}{\mathbf{X}}^{\mathbf{\prime}}{\mathbf{R}}^{-1}\mathbf{X}& {\mathbf{0}} & {\mathbf{X}}^{\mathbf{\prime}}{\mathbf{R}}^{-1}\mathbf{Z}\\ {\mathbf{0}} & {\upsigma }_{\text{u}}^{-2}\left({\upgamma }^{-1}\left(1-\frac{\upgamma }{2}\right)+{\mathbf{1}}^{\mathbf{\prime}}{\mathbf{A}}^{-1}{\mathbf{1}}\right)& -{\mathbf{1}}^{\mathbf{\prime}}{\mathbf{A}}^{-1}{\upsigma }_{\text{u}}^{-2}\\ {\mathbf{Z}}^{\mathbf{\prime}}{\mathbf{R}}^{-1}\mathbf{X}& -{\mathbf{A}}^{-1}{\mathbf{1}}{\upsigma }_{\text{u}}^{-2}& {\mathbf{Z}}^{\mathbf{\prime}}{\mathbf{R}}^{-1}\mathbf{Z}+{\mathbf{A}}^{-1}{\upsigma }_{\text{u}}^{-2}\end{array}\right),$$

which thanks to the factorization of ${\left(1-\frac{\upgamma }{2}\right)}^{-1}$ is in terms of ${\upsigma }_{\text{u}}^{-2}$, not of ${\upsigma }_{\text{u-related}}^{-2}$, with inverse $${\mathbf{M}}^{-1}=\left(\begin{array}{ccc}{\mathbf{M}}^{11}& {\mathbf{M}}^{12}& {\mathbf{M}}^{13}\\ {\mathbf{M}}^{21}& {\mathbf{M}}^{22}& {\mathbf{M}}^{23}\\ {\mathbf{M}}^{31}& {\mathbf{M}}^{32}& {\mathbf{M}}^{33}\end{array}\right)$$. Letting ${\mathbf{C}}^{22}={\left({\upsigma }_{u}^{-2}{\mathbf{A}}^{-1}+{\mathbf{Z}}^{\mathbf{\prime}}{\mathbf{R}}^{-1}\left(\mathbf{I}-{\mathbf{P}}_{\mathbf{X}}\right)\mathbf{Z}\right)}^{-1}$, i.e., the prediction error variance of the breeding values under a regular animal model:

$${\mathbf{M}}^{22}={\left({\mathbf{M}}_{22}-\left(\begin{array}{cc}{\mathbf{M}}_{21}& {\mathbf{M}}_{23}\end{array}\right){\left(\begin{array}{cc}{\mathbf{M}}_{11}& {\mathbf{M}}_{13}\\ {\mathbf{M}}_{31}& {\mathbf{M}}_{33}\end{array}\right)}^{-1}\left(\begin{array}{c}{\mathbf{M}}_{12}\\ {\mathbf{M}}_{32}\end{array}\right)\right)}^{-1}$$

13 $$={\left({\upsigma }_{\text{u}}^{-2}\left({\upgamma }^{-1}\left(1-\frac{\upgamma }{2}\right)+{\mathbf{1}}^{\mathbf{\prime}}{\mathbf{A}}^{-1}{\mathbf{1}}\right)-{\mathbf{1}}^{\mathbf{\prime}}{\mathbf{A}}^{-1}{\mathbf{C}}^{22}{\mathbf{A}}^{-1}{\mathbf{1}}{\upsigma }_{\text{u}}^{-4}\right)}^{-1}.$$

By the mixed model property derived before, ${\mathbf{C}}^{22}{\mathbf{A}}^{-1}{\mathbf{1}}{\upsigma }_{\text{u}}^{-4}={\mathbf{1}}{\upsigma }_{\text{u}}^{-2}$. Thus:

$${\mathbf{M}}^{22}={\left({\upsigma }_{\text{u}}^{-2}\left({\upgamma }^{-1}\left(1-\frac{\upgamma }{2}\right)+{\mathbf{1}}^{\mathbf{\prime}}{\mathbf{A}}^{-1}{\mathbf{1}}\right)-{\mathbf{1}}^{\mathbf{\prime}}{\mathbf{A}}^{-1}{\mathbf{1}}{\upsigma }_{\text{u}}^{-2}\right)}^{-1}$$

14 $$={\left({\upsigma }_{\text{u}}^{-2}{\upgamma }^{-1}\left(1-\frac{\upgamma }{2}\right)\right)}^{-1}={\upsigma }_{\text{u}}^{2}\frac{2\upgamma }{2-\upgamma }.$$

Hence, the prediction error variance of the metafounder effect is ${\upsigma }_{\text{u}}^{2}\frac{2\upgamma }{2-\upgamma }$. Moreover, its prediction error variance is equal to its variance as seen from Eq. (11), since ${{\upsigma }_{\text{u}}^{2}\upgamma \left(1-\frac{\upgamma }{2}\right)}^{-1}={\upsigma }_{\text{u}}^{2}\frac{2\upgamma }{2-\upgamma }$.

Then, for the animal effect block:

15 $${\mathbf{M}}^{33}={\left({{\mathbf{C}}^{22}}^{-1}-{\mathbf{A}}^{-1}{\mathbf{11}}^{\mathbf{\prime}}{\mathbf{A}}^{-1}{k}^{-1}\right)}^{-1},$$

where $k={\upsigma }_{\text{u}}^{2}\left({\upgamma }^{-1}\left(1-\frac{\upgamma }{2}\right)+{\mathbf{1}}^{\mathbf{\prime}}{\mathbf{A}}^{-1}{\mathbf{1}}\right)$. Applying a variant of the Sherman–Morrison formula ([42]; formula 14) to Eq. (15):

16 $${\mathbf{M}}^{33}={\mathbf{C}}^{22}+\frac{{\mathbf{C}}^{22}{\mathbf{A}}^{-1}{\mathbf{11}}^{\mathbf{\prime}}{\mathbf{A}}^{-1}{\mathbf{C}}^{22}{k}^{-1}}{1-{\mathbf{1}}^{\mathbf{\prime}}{\mathbf{A}}^{-1}{\mathbf{C}}^{22}{\mathbf{A}}^{-1}{\mathbf{1}}{k}^{-1}}.$$

Using the mixed model property:

$${\mathbf{M}}^{33}={\mathbf{C}}^{22}+\frac{{\upsigma }_{u}^{4} {k}^{-1}}{1-{\mathbf{1}}^{\mathbf{\prime}}{\mathbf{A}}^{-1}{\mathbf{1}}{ {\upsigma }_{u}^{2} k}^{-1}} {\mathbf{11}}^{\mathbf{\prime}}$$

$$={\mathbf{C}}^{22}+\frac{{\upsigma }_{u}^{4}}{k-{\mathbf{1}}^{\mathbf{\prime}}{\mathbf{A}}^{-1}{\mathbf{1}}{\upsigma }_{u}^{2}}{\mathbf{11}}^{\mathbf{\prime}}={\mathbf{C}}^{22}+\frac{{\upsigma }_{u}^{4}}{{\upsigma }_{\text{u}}^{2}\left({\upgamma }^{-1}\left(1-\frac{\upgamma }{2}\right)+{\mathbf{1}}^{\mathbf{\prime}}{\mathbf{A}}^{-1}{\mathbf{1}}\right)-{\mathbf{1}}^{\mathbf{\prime}}{\mathbf{A}}^{-1}{\mathbf{1}}{\upsigma }_{u}^{2}}{\mathbf{11}}^{\mathbf{\prime}}$$

17 $$={\mathbf{C}}^{22}+\frac{2\upgamma {\upsigma }_{u}^{2}}{2-\upgamma }{\mathbf{11}}^{\mathbf{\prime}}=\text{Var}\left(\widehat{{\mathbf{u}}}-{\mathbf{u}}\right)+\text{Var}\left({\widehat{u}}_{mf}-{u}_{mf}\right){\mathbf{11}}^{\mathbf{\prime}}.$$

Thus, the prediction error variance of the breeding values in a model with one metafounder is equal to the prediction error variance of the breeding values in a model without metafounders plus the prediction error variance of the metafounder effect.

Lastly, using the mixed model property:

18 $${\mathbf{M}}^{32}={\mathbf{C}}^{22}{\mathbf{A}}^{-1}{\mathbf{1}}{\upsigma }_{u}^{4}\frac{2\upgamma }{2-\upgamma }={\mathbf{1}}{\upsigma }_{u}^{2}\frac{2\upgamma }{2-\upgamma }.$$

Hence, the prediction error covariance between an animal effect and the metafounder effect is equal to the prediction error variance of the metafounder.

Using Eqs. (14) and (17) and $\text{Var}\left({\widehat{u}}_{i}-{u}_{i}\right)={\mathbf{A}}_{ii}\left(1-{rel}_{i}\right)$, the reliability of an animal under a model with one metafounder is:

$${rel}_{i}^{*}=1-\frac{\text{Var}\left({\widehat{u}}_{i}-{u}_{i}\right)+\text{Var}\left({\widehat{u}}_{mf}-{u}_{mf}\right)}{{\mathbf{A}}_{ii}{\upsigma }_{u}^{2}+\upgamma {\left(1-\frac{\upgamma }{2}\right)}^{-1} {\upsigma }_{u}^{2}}$$

19 $$=1-\frac{\left(2-\upgamma \right){\mathbf{A}}_{ii}\left(1-{rel}_{i}\right)+2\upgamma }{\left(2-\upgamma \right){\mathbf{A}}_{ii}+2\upgamma }=\frac{\left(2-\upgamma \right){\mathbf{A}}_{ii}}{\left(2-\upgamma \right){\mathbf{A}}_{ii}+2\upgamma } {rel}_{i}.$$

Given the range of $\upgamma$, ${rel}_{i}^{*}<{rel}_{i}$.

The reliability of the contrast between the $i$th animal and the metafounder $\left(re{l}_{{c}_{i}}\right)$ is:

$$re{l}_{{c}_{i}}=1-\frac{\text{Var}\left(\left({\widehat{u}}_{i}^{*}-{u}_{i}^{*}\right)-\left({\widehat{u}}_{mf}-{u}_{mf}\right)\right)}{\text{Var}\left({u}_{i}^{*}-{u}_{mf}\right)}$$

$$=1-\frac{\text{Var}\left({\widehat{u}}_{i}^{*}-{u}_{i}^{*}\right)+\text{Var}\left({\widehat{u}}_{mf}-{u}_{mf}\right)-2\text{ cov}\left(\left({\widehat{u}}_{i}^{*}-{u}_{i}^{*}\right),\left({\widehat{u}}_{mf}-{u}_{mf}\right)\right)}{\text{Var}\left({u}_{i}^{*}\right)+\text{Var}\left({u}_{mf}\right)-2 \text{cov}\left({u}_{i}^{*},{u}_{mf}\right)}$$

$$=1-\frac{{\mathbf{M}}_{ii}^{22}+{\mathbf{M}}^{33}-2 {\mathbf{M}}_{i}^{23}}{\left({\mathbf{A}}_{{\upgamma }_{ii}}+{\mathbf{A}}_{{\upgamma }_{mf,mf}}-2 {\mathbf{A}}_{{\upgamma }_{i,mf}}\right){\upsigma }_{u}^{2}}$$

$$=1-\frac{{\mathbf{C}}_{ii}^{22}+\frac{2\upgamma {\upsigma }_{u}^{2}}{2-\upgamma }+\frac{2\upgamma {\upsigma }_{u}^{2}}{2-\upgamma }-2 \frac{2\upgamma {\upsigma }_{u}^{2}}{2-\upgamma }}{\left({\mathbf{A}}_{ii}{\upsigma }_{u}^{2}+\upgamma {\left(1-\frac{\upgamma }{2}\right)}^{-1} {\upsigma }_{u}^{2}\right)+\left(\upgamma {\left(1-\frac{\upgamma }{2}\right)}^{-1}\right){\upsigma }_{u}^{2}-2\left(\upgamma {\left(1-\frac{\upgamma }{2}\right)}^{-1}\right){\upsigma }_{u}^{2}}$$

20 $$=1-\frac{{\mathbf{C}}_{ii}^{22}}{{\mathbf{A}}_{ii}{\upsigma }_{u}^{2}}=1-\frac{\text{Var}\left({\widehat{u}}_{i}-{u}_{i}\right)}{\text{Var}\left({u}_{i}\right)}= {rel}_{i}.$$

Thus, the reliability of the contrast ($re{l}_{{c}_{i}}$) in a model with one metafounder and the reliability of the regular animal model (${rel}_{i}$) are identical.

For multiple-trait models, the necessary mixed-model property is $\left(\left(\mathbf{K}\otimes {\mathbf{G}}_{0}\right){{\mathbf{C}}^{22}}^{-1}\right)\left({\mathbf{1}}\otimes \mathbf{I}\right)=({\mathbf{1}}\otimes \mathbf{I})$, where $\mathbf{K}$ is the covariance matrix for random effects and ${\mathbf{G}}_{0}$ is the covariance matrix between traits. Then, Eq. (20) holds because it can be proved that the results for Eqs. (14), (17), and (18) are ${\mathbf{G}}_{0}\frac{2\upgamma }{2-\upgamma }$, ${\mathbf{C}}^{22}+{\mathbf{11}}^{\mathbf{\prime}}\otimes {\mathbf{G}}_{0}\frac{2\upgamma }{2-\upgamma }$, and ${\mathbf{1}}\otimes {\mathbf{G}}_{0}\frac{2\upgamma }{2-\upgamma }$, respectively.
